# Antileishmanial Potential of Tropical Rainforest Plant Extracts

**DOI:** 10.3390/medicines1010032

**Published:** 2014-11-19

**Authors:** Lianet Monzote, Abel Piñón, William N. Setzer

**Affiliations:** 1 Parasitology Department, Institute of Tropical Medicine “Pedro Kouri”, 10400 Havana, Cuba; E-Mail: monzote@ipk.sld.cu; 2 Department of Chemistry, University of Alabama in Huntsville, Huntsville, AL 35899, USA; E-Mail: wsetzer@chemistry.uah.edu

**Keywords:** leishmaniasis, *Leishmania amazonensis*, Abaco island, Bahamas, Matabeleland, Zimbabwe, Monteverde, Costa Rica, Far North Queensland, Australia

## Abstract

A total of 115 different plant extracts from our collection, representing 96 plant species, have been evaluated for *in vitro* antileishmanial activity against *L. amazonensis* promastigotes. In addition, the extracts were screened for cytotoxic activity against BALB/c mouse macrophages in order to assess a selectivity index. Crude extracts that showed a selectivity index (*CC*_50_ for macrophage / *IC*_50_ for promastigotes) ≥ 5 or with *IC*_50_ < 12.5 μg/mL against promastigotes, a total of 28 extracts, were further screened for anti-amastigote activity. A total of 25 extracts showed promising activity against *L. amazonensis* promastigotes with low cytotoxic activity. Ten of these extracts showed selectivity indices, (*CC*_50_ for macrophages / *IC*_50_ for amastigotes) greater than 10 and are considered “hits”, worthy candidates for further phytochemical exploration: *Conostegia xalapensis* methanol bark extract, *Endiandra palmerstonii* bark extract, *Eugenia monteverdensis* acetone bark extract, *Eugenia* sp. “fine leaf” acetone bark extract, *Exothea paniculata* chloroform bark extract, *Mallotus paniculatus* ethanol bark extract, *Matelea pseudobarbata* ethanol extract, *Quercus insignis* ethanol bark extract, *Sassafras albidum* dichloromethane bark extract, and *Stemmadenia donnell-smithii* acetone bark extract.

## 1. Introduction

Leishmaniasis is a collection of chronic infectious diseases caused by different species of parasitic *Leishmania* protozoa and are transmitted by sandflies (*Phlebotomus* spp. and *Lutzomyia* spp.) [1,2]. The disease, considered to be a “neglected disease”, currently affects around 12 million people, with nearly 350 million people at risk of infection around the world. The clinical forms of leishmaniasis have been classified as cutaneous, mucocutaneous, or visceral. Additionally, global climate change will likely affect the geographical range of *Leishmania* infections in North America [3] and Europe [4]. Indeed, *Phlebotomus* sandfly distribution in Europe has shifted as far north as Germany [5] while *Lutzomyia* sandflies are now found as far north as Ohio [6], Maryland, and Delaware [7].

Current proven chemotherapy includes the pentavalent antimonials meglumine antimoniate (glucantime) and sodium stibogluconate (pentostam) [8], miltefosine, amphotericin B, or pentamidine [9]. However, all of these treatments are associated with undesirable side effects, induction of parasite resistance, and relatively high cost in developing countries. Due to the limitations of current chemotherapeutic regimes, along with the absence of suitable vaccines, there is a persistent need for alternative and readily available chemotherapies for treatment of leishmaniasis.

Natural products have overwhelmingly contributed to the pharmacopeia of the world and continue to provide new and effective anti-infective agents [10,11,12]. Recently, several reviews have appeared demonstrating the potential of higher plants and phytochemicals as treatment options for leishmaniasis [13,14,15,16,17,18,19]. In this current work, we present the *in vitro* screening of 115 different extracts from higher plants (96 species) collected from rainforests of north Queensland, Australia, Abaco Island, Bahamas, Costa Rica, southeastern U.S., and Zimbabwe, against *Leishmania amazonensis*.

## 2. Experimental Section 

### 2.1. Plant Materials and Reference Drugs

Plant materials from 96 species of plants (Table 1) were collected and extracted as previously described [20,21,22]. The crude extracts were dissolved in dimethylsulfoxide (DMSO) at 20 mg/mL. The reference drugs amphotericin B (Imefa, Havana, Cuba) and pentamidine (Richet, Buenos Aires, Argentina) were also used.

### 2.2. Parasite Cultures

*L. amazonensis* (MHOM/77BR/LTB0016) was kindly provided by the Department of Immunology, Oswaldo Cruz Foundation (FIOCRUZ), Brazil. Parasites were routinely isolated from mouse lesions and maintained as promastigotes at 26 °C in Schneider’s medium (SIGMA, St. Louis, MO, USA) containing 10% heat-inactivated fetal bovine serum (HFBS) (SIGMA, St. Louis, MO, USA), 100 μg of streptomycin/mL, and 100 U penicillin/mL. The parasites were not used after the tenth passage.

### 2.3. Anti-Promastigote Assay

Schneider’s medium (50 µL) was distributed in each well of a 96-well plate. In the first well of each lane, 48 µL of medium and 2 µL of tested extracts were added. The extract solutions were serially diluted (1:1) down each lane of the 96-well plate with medium (removing 50 μL of test solution and diluting with 50 μL medium). Then, 50 µL of exponentially growing cells at 2 × 10^5^ promastigotes/mL were added to each well to give final concentrations ranging from 12.5 to 200 μg/mL. After an incubation of 72 hours at 26 °C, 20 µL of 3-[4,5-dimethylthiazol-2-yl]-2,5-diphenyltetrazolium bromide (MTT) (SIGMA, St. Louis, MO, USA) was added. MTT solutions were prepared at 5 mg/mL in PBS, filtered and sterilized just prior to use. After incubation for an additional 4 h, the formazan crystals were dissolved by addition of 100 μL of DMSO. The optical density was determined using a spectrophotometer (Sirio S Reader, 2.4-0, Italy), at a test wavelength of 560 nm and a reference wavelength of 630 nm [23,24] and median inhibitory concentrations (*IC*_50_) were calculated.

### 2.4. Cytotoxicity Assay

The cytotoxic median concentration (*CC*_50_) of the extracts was determined on mouse peritoneal macrophages, which are the host cells for the amastigote form of the parasite, aiming to investigate macrophage toxicity caused by the extracts [23]. Resident macrophages were collected from peritoneal cavities of normal BALB/c mice in ice-cold RPMI 1640 medium (SIGMA, St. Louis, MO, USA) supplemented with antibiotics, and seeded at 30,000 cells/well. The cells were incubated for 2 h at 37 °C in 5% CO_2_. Non-adherent cells were removed by washing with phosphate-buffered saline (PBS), and then 2 μL of extract/DMSO solutions were added to 98 μL medium with 10% HFBS and antibiotics. Then, five two-fold dilutions were carried out taking 50 µL each time and adding an additional 50 µL of medium to give test concentrations of the extracts ranging from 12.5 to 200 μg/mL. The cells were incubated with the test solutions for 72 h. Macrophages treated with DMSO were included as controls. The cytotoxicity was determined using the colorimetric assay with MTT as previously described; 15 μL MTT solution added to each well. After incubation for an additional 4 h the formazan crystals were dissolved by addition of 100 μL of DMSO and the optical density was determined. 

### 2.5. Selectivity Index

The selectivity index (SI) ratio (*CC*_50_ for macrophage / *IC*_50_ for promastigotes) was used to compare the toxicity of the extracts for murine macrophages and the activity against *Leishmania* promastigotes. Extracts with a SI ≥ 5 or with *IC*_50 _< 12.5 μg/mL against promastigotes were selected for further anti-amastigote determination.

### 2.6. Anti-Amastigote Assay

The peritoneal macrophages were harvested and plated at 10^6^ cells/mL in 24-well Lab-Tek plates (Costar^®^, Washington, DC, USA) and incubated at 37 °C under an atmosphere of 5% CO_2_ for 2 h. Non-adherent cells were removed by washing with PBS. Stationary-phase of *L. amazonensis* promastigotes were added at a 4:1 parasite/macrophage ratio and the cultures were incubated for an additional 4 h. The cell monolayers were washed three times with PBS to remove free parasites. Then, 1990 μL of RPMI completed medium and 10 μL of the different extracts dissolved in DMSO were added to each well. Four two-fold dilutions were carried out taking 1000 µL each time to give test concentrations of the extracts ranging from 12.5 to 100 μg/mL, in duplicate cultures, incubating for an additional 48 h [25]. The parasites were then fixed in absolute methanol, stained with Giemsa, and examined under light microscopy. The number of intracellular amastigotes was determined by counting the amastigotes in 25 macrophages per sample, and the results were expressed as percent of reduction of the infection rate in comparison to that of the controls. The infection rates were obtained by multiplying the percentage of infected macrophages by the number of amastigotes per infected macrophages [26] and the *IC*_50_ value was determined. A second SI was calculated: *CC*_50_ for macrophages / *IC*_50_ for intracellular amastigotes (see Table 1).

### 2.7. Statistical Analyses

The *IC*_50_ of the tested extracts and reference drugs against *L. amazonensis* promastigotes and amastigotes and the *CC*_50_ on peritoneal macrophages from BALB/c mice were obtained from dose-response curves fit to the data by means of the linear equation model using the STATISTICA for Windows Program (Release 4.5, StatSoft, Inc., Tulsa, OK, USA, 1993). The evaluations were performed in triplicate and the results are expressed as means ± standard deviations.

## 3. Results and Discussion 

A total of 115 different plant extracts from our collection, representing 96 plant species, have been screened for *in vitro* antileishmanial activity against *L. amazonensis*. In addition, the extracts were screened for cytotoxic activity against BALB/c mouse macrophages in order to assess a selectivity index. The antileishmanial and cytotoxic activities are summarized in Table 1. A total of 18 extracts showed activity against *L. amazonensis* promastigotes with *IC*_50_ values < 12.5 μg/mL. An additional 25 extracts showed antileishmanial activities ranging from *IC*_50_ 17.4 μg/mL to 63.4 μg/mL. Of the plant extracts that were active against *L. amazonensis* promastigotes, 18 were non-toxic to BALB/c mouse macrophages (*CC*_50_ > 200 μg/mL). In addition, 10 others were only slightly toxic to the mouse macrophages, showing selectivity indices (*CC*_50_ for macrophage / *IC*_50_ for promastigotes) ≥ 5, and were considered for further testing against *L. amazonensis* amastigotes. Additional screening against *L. amazonensis* amastigotes revealed ten extracts with *IC*_50_ ≤ 21.0 μg/mL against the parasites and non-toxic to the mouse macrophages (*CC*_50_ > 200 μg/mL). That is, the selectivity indices (*CC*_50_ for macrophage / *IC*_50_ for amastigotes) for these extracts were all > 10. It is generally considered that for plant extracts showing SI > 10, the antiprotozoal activity is not due to general cytotoxicity and are promising extracts for further phytochemical analysis [27,28,29]. 

The methanol bark extract of *Conostegia xalapensis* (Melastomataceae) showed good leishmanicidal activity (*IC*_50_ < 12.5 μg/mL against both promastigotes and amastigotes), but was neither cytotoxic to BALB/c mouse macrophages nor human tumor cells [21]. Thus, this plant extract shows excellent selectivity and apparently low acute toxicity. To our knowledge, no phytochemical work has been carried out on this plant; it is not known what compounds may be responsible for the antileishmanial activity. Although the dichloromethane bark extract of *Quercus insignis* (Fagaceae) was inactive, the ethanol bark extract exhibited notable antileishmanial activity against *L. amazonensis* promastigotes and amastigotes (*IC*_50_ = 17.8 and 21.0 μg/mL, respectively). *Q. insignis* ethanol bark extract showed no *in vitro* toxicity to mouse macrophage cells or human tumor cells [21]. The phytochemistry of this tree has not been examined.

Interestingly, both the chloroform and methanol bark extracts of *Exothea paniculata* (Sapindaceae) from Monteverde, Costa Rica, showed good activity against *L. amazonensis* with little cytotoxicity on BALB/c mouse macrophages, but neither the acetone bark extract nor the methanol bark extract of *E. paniculata* from Abaco Island, Bahamas was leishmanicidal. The chloroform extract of *E. paniculata* from Monteverde showed the most promise with *IC*_50_ < 12.5 μg/mL against both the promastigote and amastigote forms of *L. amazonensis*, but non-toxic to macrophage cells. There have been no reported studies on the phytochemical composition of *E. paniculata*.

An ethanol extract of the aerial parts of *Matelea pseudobarbata* (Apocynaceae) was shown to be non-cytotoxic to mouse macrophages, but notably leishmanicidal against *L. amazonensis* promastigotes and amastigotes (*IC*_50_ < 12.5 μg/mL). Additionally, this extract was non-cytotoxic to Hep-G2, MCF-7, MDA-MB-231, and PC-3 tumor cell lines [21]. Apparently little else is known about this vine. The acetone bark extract of *Stemmadenia donnell-smithii* (Apocynaceae) was also antileishmanial (*IC*_50_ < 12.5 μg/mL) and non-toxic to the mouse macrophage. *S. donnell-smithii* is cytotoxic to human tumor cells, however [21]. A decoction of *S. donnell-smithii* bark is used as a traditional medicine in Guatemala to treat malaria [30]. Several indole alkaloids have been isolated from *S. donnell-smithii*, including (+)-quebrachamine, voacangine, isovoacangine, voacamine, tabernanthine, ibogamine, and stemmadine [31] and may be responsible for the leishmanicidal activity of *S. donnell-smithii*. An extract of *Tabernaemontana catharinensis*, rich in voacangine (53%) has shown antileishmanial activity against *L. amazonensis* [32]. Voacamine has shown antiplasmodial activity [33] and a molecular docking study revealed voacamine to dock strongly to *Leishmania N*-myristoyl transferase as a molecular target [34].

Two different species of *Eugenia* (Myrtaceae) from Monteverde, Costa Rica, showed good antileishmanial activity. Thus, the acetone bark extracts of *E. monteverdensis* and *Eugenia* sp. “fine leaf” were active against *L. amazonensis* promastigotes (*IC*_50_ = 23.9 and < 12.5 μg/mL, respectively), while neither was cytotoxic to BALB/c mouse macrophages (*CC*_50_ > 200 μg/mL). Furthermore, *E. monteverdensis* acetone bark extract was inactive against MCF-7 and Hs 578T human breast tumor cells (unpublished results from this laboratory), and *Eugenia* “fine leaf” acetone bark extracts was inactive on several human tumor cell lines (Hep-G2 MDA-MB-231, Hs 578T, and 5637) [21]. Consistent with these results, the ethanol bark extracts of *E. austin-smithii* and a *Eugenia* sp. from the Alberto Manuel Brenes Biological Reserve, Costa Rica, showed antileishmanial activity [35]. The acetone bark extract of *E. austin-smithii* has been found to be cytotoxic to Hs 578T, 5637, and BHK cells [21]. Both the hexane and methanol fruit extracts of *E. umbelliflora* were leishmanicidal to *L. amazonensis* and *L. braziliensis* promastigotes [36]. *E. uniflora* has been screened for antileishmanial activity. The ethanol leaf extract was marginally active against *L. braziliensis* promastigotes (65% inhibition at 100 μg/mL) [37], but inactive against *L. amazonensis* or *L. chagasi* promastigotes [38]. The leaf essential oil of *E. uniflora* showed good antileishmanial activity against both promastigotes and amastigotes of *L. amazonensis* (*IC*_50_ = 3.04 and 1.92 μg/mL, respectively) [39], but *E. uniflora* bark essential oil was inactive against *L. donovani* promastigotes [40]. 

The bark of *Endiandra palmerstonii* (Lauraceae) from far north Queensland was extracted using a chloroform/ethanol mixed solvent [22]. The crude extract showed promising antileishmanial activity (*IC*_50_ < 12.5 μg/mL on promastigotes and 18.7 μg/mL on amastigotes) and no cytotoxicity on BALB/c mouse macrophages (*CC*_50_ > 200 μg/mL). The extract was also non-cytotoxic to Hep-G2 human liver tumor cells [22]. There have been no phytochemical investigations reported for this tree species.

Two different bark extracts of *Mallotus* (Euphorbiaceae) from far north Queensland, Australia, *M. mollisimus* (CHCl_3_/EtOH mixed solvent bark extract) and *M. paniculatus* (EtOH bark extract), showed promising antileishmanial activity coupled with selectivity. *M. paniculatus* ethanol bark extract was the more promising with *IC*_50_ < 12.5 μg/mL on both promastigotes and amastigotes, and no cytotoxicity to macrophage cells. Previous screening for cytotoxicity against a panel of human tumor cell lines revealed *M. paniculatus* ethanol bark extract to be non-cytotoxic to Hep-G2, MDA-MB-231, Hs 578T, and 5673 cells [22]. Thus, this plant extract is apparently non-toxic to mammalian cells while remarkably toxic to the parasite. The triterpenoid 29-nor-3,22-hopanedione has been isolated from the stem bark of *M. paniculatus*, but no bioactivities were reported for this compound [41]. *M. oppositifolius* leaves are used in traditional medicine in the Ivory Coast to treat intestinal helminths, but the leaf extracts were inactive against *L. donovani* promastigotes [42].

Both the dichloromethane bark extract and the ethyl acetate bark extract of *Sassafras albidum* (Lauraceae), collected from north Alabama, showed notable antileishmanial activities against promastigotes (*IC*_50_ < 12.5 and 19.4 μg/mL, respectively), and the dichloromethane extract had a median inhibitory concentration of 20.5 μg/mL against amastigotes of *L. amazonensis*. In addition, the extracts were non-toxic to macrophage cells. Although the essential oil compositions of *S. albidum* have been investigated [43,44], apparently the non-volatile bark components have not. *S. albidum* bark oil is rich in α-pinene, β-pinene, 1,8-cineole, and α-terpineol [44]. Many essential oils and components have shown antiprotozoal activities [45], including α-pinene [46] and α-terpineol [47].

The acetone bark extract of *Acacia choriophylla* (Fabaceae) from Abaco Island, Bahamas, showed a selectivity index >3. The dichloromethane bark extract of the same tree showed similar activity. Both of these crude extracts had been previously screened for *in vitro* cytotoxic activity against 5637 bladder tumor and Hs 578T breast tumor cells (unpublished results from our laboratory) and were non-cytotoxic, so neither seems to be acutely toxic to human cells. The phytochemistry of this tree has not been examined. 

The acetone bark extract of *Tabebuia bahamensis* (Bignoniaceae) was active against *L. amazonensis* promastigotes and amastigotes with *IC*_50_ values of 17.4 and 23.8 μg/mL, respectively. This bark extract was screened for cytotoxic activity against a panel of human tumor cell lines, including SK-Mel-38 (melanoma), Hep-G2 (hepatocellular carcinoma), MDA-MB-231 (mammary adenocarcinoma), and 5637 (bladder carcinoma), and was found to be inactive against all cell lines screened (unpublished results from our laboratory). *T. umbellata* is used traditionally in Bahia, Brazil to treat cutaneous leishmaniasis (*L. braziliensis*) infections [48]. Likewise, *T. serratifolia* bark is used in Peru to treat leishmaniasis, and *T. serratifolia* chloroform bark extract did show *in vitro* activity against *L. infantum* promastigotes [49]. Naphthoquinones from *T. avellanedae* [50] and *T. serratifolia* [49] have shown antileishmanial activity against *L. major* and *L. infantum*, respectively. Thus, the antileishmanial activity of *T. bahamensis* bark extract may also be due to naphthoquinones.

The bark resin from *Agathis atropurpurea* (Araucariaceae) exhibited good antileishmanial activity (*IC*_50_ < 12.5 and 19.3 μg/mL, respectively against *L. amazonensis* promastigotes and amastigotes) along with weak cytotoxic activity against BALB/c mouse macrophage cells. This resin had shown cytotoxic activity against SK-MEL-28 human melanoma cells, but was inactive against the Hep-G2, MDA-MB-231, Hs 578T, MCF-7, and PC-3 cell lines [22]. The resin is rich in diterpenoids, including agatholic acid [51]. 

Four species of *Melicope* (Rutaceae) from north Queensland, Australia, were screened for antileishmanial activity. *M. broadbentiana* and *M. vitiflora* bark extracts were inactive, but *M. jonesii* chloroform bark extract and *M. rubra* ethanol bark extract showed promising activity against *L. amazonensis* promastigotes. Further screening revealed no selectivity for *L. amazonensis* amastigotes over mouse macrophages, however. Both *M. jonesii* and *M. rubra* bark extracts showed *in vitro* cytotoxicity to human tumor cells [22]. Although the leaf essential oils of *M. jonesii* and *M. rubra* have been investigated [52], there are no reports on the bark phytochemistry. The chloroform bark extract of *Polyosma alangiacea* (Escalloniaceae) from far north Queensland, Australia was somewhat leishmanicidal (*IC*_50_ = 60.9 μg/mL) against promastigotes, but non-cytotoxic to mammalian cells, either mouse macrophages or human tumor cells [22]. The phytochemistry of *P. alangiacea* bark has not been reported.

The chloroform bark extract of an, as yet, undescribed species of *Myrcianthes* “black fruit” (Myrtaceae) from Monteverde, Costa Rica, does show promise with leishmanicidal *IC*_50_ of < 12.5 and 18.3 μg/mL, respectively, against promastigotes and amastigotes of *L. amazonensis*. The aerial parts of the liana *Ruyschia phylladenia* (Marcgraviaceae), collected from Monteverde, Costa Rica, were extracted with dichloromethane. The extract inhibited *L. amazonensis* promastigotes (*IC*_50_ < 12.5 μg/mL) and amastigotes (*IC*_50_ = 22.0 μg/mL). Although this extract was cytotoxic to human tumor cells [21] no cytotoxicity on BALB/c mouse macrophages was observed.

The dichloromethane bark extract of *Cupania glabra* (Sapindaceae), collected from Monteverde, Costa Rica, had shown remarkable *in vitro* cytotoxic activity against several human tumor cell lines, which was attributed to the fatty alcohol glycoside cupanioside [53]. The ethanol bark extract of this tree, on the other hand, was devoid of cytotoxic activity [21], and in this work, the ethanol extract was leishmanicidal (*IC*_50_ = 17.6 and 27.0 μg/mL on promastigotes and amastigotes, respectively) and non-toxic to BALB/c mouse macrophages. Consistent with these results, the crude methanol bark extract of *C. dentata* from the Yucatan peninsula of Mexico showed *in vitro* antileishmanial activity against *L. mexicana* promastigotes (*IC*_50_ = 13 μg/mL) [54]. Interestingly, the crude hexane bark extract of *C. cinerea* showed *in vitro* leishmanicidal activity against *L. donovani* axenic amastigotes [29], possibly attributable to the diterpene glycoside cupacinoside [55]. Similarly, the hexane leaf extract of *C. vernalis* was active against *L. donovani* promastigotes [56]. Extracts of *C. macrophylla* from Costa Rica, on the other hand, were inactive against *Leishmania* promastigotes [35].

The crude ethanol bark extract of *Diospyros digyna* (Ebenaceae) showed *in vitro* antileishmanial activity against *L. amazonensis* promastigotes and amastigotes with *IC*_50_ = 25.0 and 29.2 μg/mL, respectively. Additionally, *D. digyna* extract was not cytotoxic to either BALB/c mouse macrophages or MCF-7, UACC-257, MDA-MB-231, or M-14 human tumor cells (unpublished results from this laboratory). Diospyrin, a bis-naphthoquinone isolated from *D. montana*, has shown *in vitro* activity against *L. donovani* promastigotes [57] and *L. major* promastigotes [58]. This compound has been shown to be a topoisomerase I inhibitor of *L. donovani* [59] and initiates apoptosis in *L. donovani* [60]. Antileishmanial bis-naphthoquinones have also been isolated and characterized from *D. assimilis* chloroform root extract from India (*in vitro* assay against *L. donovani* axenic amastigotes) [61] and *D. burmanica* methanol wood extract from Myanmar (*in-vitro* assay against *L. major* promastigotes) [62].

The crude ethanol bark extract of *Erythrina lanceolata* (Fabaceae) showed promising antileishmanial activity (*IC*_50_ < 12.5 μg/mL against promastigotes) without cytotoxic activity against BALB/c mouse macrophages or a number of human tumor cell lines (Hep-G2, MDA-MB-231, MCF-7, Hs 578T, PC-3, SK-MEL-28), or baby hamster kidney (BHK) cells [21]. In contrast, the crude dichloromethane bark extract was not leishmanicidal but cytotoxic to all cells tested [21]. The hydroalcoholic extract of *E. speciosa* from Brazil was tested for antileishmanial activity against both *L. amazonensis* promastigotes and amastigotes, but was inactive [63]. Likewise, the methanol wood extract of *E. suberosa* from Myanmar was inactive against *L. major* promastigotes [64], and the ethanol bark extract of *E. variegata* from New Caledonia was inactive against *L. donovani* promastigotes [65].

The acetone bark extract of *Inga sierrae* (Fabaceae) from Monteverde, Costa Rica, showed leishmanicidal activity (*IC*_50_ = 25.7 μg/mL) with reduced cytotoxic activity (*CC*_50_ = 130.6 μg/mL). Several *Inga* species are used in traditional medicine to treat leishmaniasis. *I. edulis* leaves are used by the Kichwa in Ecuador [29] while *I. edulis* bark is used by the Wayãpi people of French Guiana [66] to treat leishmaniasis. The Chachi people of Ecuador also use *I. oerstediana* leaves and bark [29] and *I. bourgoni* bark is also used by the Wayãpi [66] to treat leishmaniasis.

The methanol bark extract from *Pappea capensis* (Sapindaceae) collected in Matabeleland, Zimbabwe, also showed promising antileishmanial activity (*IC*_50_ < 12.5 μg/mL). *Rhynchosia resinosa* (Fabaceae) methanol root extract, from Matabeleland, Zimbabwe, did not exhibit cytotoxic activity toward BALB/c mouse macrophages, but was active against *L. amazonensis* amastigotes with an *IC*_50_ of 27.7 μg/mL. The methanol bark extract of *R. edulis* from Monteverde, Costa Rica was shown to be inactive against *L. amazonensis* (this work), while the methanol extract of the whole plant of *R. reniformis* from Karak, Pakistan was also non-leishmanicidal [67].

Both the dichloromethane and ethanol extracts of the aerial parts of *Conradina canescens* (Lamiaceae) from north Florida showed promising antileishmanial activity. The dichloromethane extract did show some cytotoxicity on BALB/c mouse macrophages (*CC*_50_ = 63.7 μg/mL) as well as MCF-7 and MDA-MB-231 human breast tumor cells (unpublished results from our laboratory). The essential oil composition of *C. canescens* has been reported [68], but there have been no reports regarding the non-volatile components. The major components in the essential oil were 1,8-cineole, camphor, α-pinene, *p*-cymene, *cis*-pinocamphone, myrtenal, myrtenol, verbenone, and myrtenyl acetate. 1,8-Cineole is inactive against *Leishmania* spp. [69] but α-pinene is active [46]. Myrtenal, camphor, and verbenone have shown antitrypanosomal activity [70].

**Table 1:** Antileishmanial (*Leishmanial amazonensis* promastigotes) and cytotoxic screening of tropical rainforest plant extracts.

Plant Name	Origin	Extract	Ref.	*L. amazonensis* promastigotes*IC*_50_ (μg/mL)	BALB/c Mouse macrophage*CC*_50_ (μg/mL)	Selectivit*y* Index (*CC*_50_/*IC*_50_)	*L. amazonensis* amastigotes *IC*_50_ (μg/mL)	Selectivity Index (*CC*_50_/*IC*_50_)
*Acacia choriophylla* Benth.	Abaco ^a^	acetone bark	b	63.4 ± 4.9	> 200	> 3	61.5 ± 4.6	> 3
*Acacia choriophylla* Benth.	Abaco	CH_2_Cl_2_ bark	b	74.1 ± 2.4	197.6 ± 1.4	3	-	-
*Acnistus arborescens* (L.) Schltdl.	Monteverde ^c^	acetone bark	[21]	> 200	36.8 ± 3.4	-	-	-
*Acronychia acronychioides* (F. Muell.) T. Hartley	Paluma ^d^	EtOH bark	[20]	> 200	47.3 ± 7.6	-	-	-
*Agathis atropurpurea* B. Hyland	F. No. Qld. ^e^	bark resin	[22]	< 12.5	118.4 ± 0.8	> 9	19.3 ± 2.5	6
*Albizia adinocephala* (Donn. Sm.) Britton & Rose ex Record	Monteverde	acetone bark	b	> 200	> 200	-	-	-
*Alchornea latifolia* Sw.	Monteverde	EtOH bark	[21]	> 200	> 200	-	-	-
*Alloxylon flammeum* P. Westpm & Crisp	F. No. Qld.	CHCl_3_/EtOH bark	[22]	> 200	55.5 ± 9.1	-	-	-
*Alphitonia petriei* Braid & C. White	Paluma	EtOH bark	[20]	> 200	> 200	-	-	-
*Alzatea verticillata* Ruiz & Pav.	Monteverde	EtOH bark	[21]	> 200	> 200	-	-	-
*Apodytes brachystylis* F. Muell.	Paluma	EtOH bark	[20]	< 12.5	< 12.5	1	-	-
*Archidendron vaillantii* (F. Muell.) F. Muell.	Paluma	EtOH bark	[20]	92.0 ± 8.1	< 12.5	0	-	-
*Ardisia compressa* Kunth	Monteverde	EtOH bark	[21]	> 200	146.2 ± 6.3	-	-
*Ardisia palmana* Donn. Sm.	Monteverde	acetone bark	[21]	143.7 ± 11.1	96.6 ± 2.6	-	-	-
*Ardisia revoluta* Kunth	Monteverde	acetone bark	[21]	> 200	70.5 ± 6.2	-	-	-
*Ardisia revoluta* Kunth	Monteverde	CH_2_Cl_2_ bark	[21]	44.0 ± 2.9	49.2 ± 1.5	1	-	-
*Ardisia solomonii* Lundell	Monteverde	CH_2_Cl_2_ bark	b	> 200	26.3 ± 2.3	-	-	-
*Ardisia solomonii* Lundell	Monteverde	CH_2_Cl_2_ bark	b	28.1 ± 2.0	< 12.5	-	-	-
*Balanops australiana* F. Muell.	Paluma	EtOH bark	[20]	49.0 ± 4.3	< 12.5	0	-	-
*Beilschmiedia* sp. “choncho blanco”	Monteverde	EtOH bark	[21]	> 200	> 200	-	-	-
*Bocconia frutescens* L.	Monteverde	MeOH bark	[21]	> 200	168.0 ± 4.5	-	-	-
*Bravaisia integerrima* (Spreng.) Standley	Monteverde	CHCl_3_ bark	[21]	76.6 ± 0.5	21.6 ± 5.0	0	-	-
*Bridelia mollis* Hutch.	Matabeleland^f^	MeOH bark	b	> 200	> 200	-	-	-
*Byrsonima crassifolia* (L.) Kunth	Monteverde	acetone bark	b	> 200	66.2 ± 0.8	-	-	-
*Cardwellia sublimis* F. Muell.	Paluma	EtOH bark	[20]	> 200	> 200	-	-	-
*Cavendishia bracteata* (Ruiz & Pav. ex J. St.-Hil.) Hoerold	Monteverde	acetone bark	b	38.4 ± 6.9	76.0 ± 5.6	2	-	-
*Cestrum megalophyllum* Dunal	Monteverde	EtOH bark	[21]	> 200	28.6 ± 1.1	-	-	-
*Chionanthus panamensis* (Standl.) Stearn	Monteverde	acetone bark	[21]	> 200	78.2 ± 6.4	-	-	-
*Conostegia xalapensis* (Bonpl.) D. Don	Monteverde	MeOH bark	[21]	< 12.5	> 200	> 16	< 12.5	> 16
*Conradina canescens* A. Gray	Florida^g^	CH_2_Cl_2_ aerial parts	b	< 12.5	63.7 ± 5.0	> 5	20.5 ± 5.0	3
*Conradina canescens* A. Gray	Florida	EtOH aerial parts	b	33.6 ± 5.9	202.8 ± 6.8	6	30.6 ± 3.9	7
*Cryptocarya corrugata* C. White & Francis	Paluma	EtOH bark	[20]	> 200	88.7 ± 5.3	-	-	-
*Cryptocarya densiflora* Blume	Paluma	EtOH bark	[20]	> 200	> 200	-	-	-
*Cupania glabra* Sw.	Monteverde	EtOH bark	[21]	17.6 ± 5.3	> 200	> 11	27.0 ± 4.5	> 7
*Daphandra repandula* (F. Muell.) F. Muell.	F. No. Qld.	CHCl_3_/EtOH bark	[22]	> 200	< 12.5	-	-	-
*Dendropanax gonatopus* (Donn. Sm.) A.C. Sm.	Monteverde	MeOH leaf	[23]	> 200	72.4 ± 7.2	-	-	-
*Diospyros digyna* Jacq.	Monteverde	EtOH bark	b	25.0 ± 2.9	152.9 ± 8.8	6	29.2 ± 1.2	5
*Diospyrus* sp. “fluted trunk”	Monteverde	EtOH bark	b	> 200	57.4 ± 3.5	-	-	-
*Drymonia conchocalyx* Hanst.	Monteverde	EtOH aerial parts	[21]	> 200	111.1	-	-	-
*Drypetes lasiogyna* F. Muell.	Paluma	EtOH bark	[20]	47.6 ± 2.2	125.8 ± 8.2	3	-	-
*Elaeodendron matabelicum* Loes.	Matabeleland	MeOH bark	b	> 200	125.7 ± 9.0	-	-	-
*Endiandra palmerstonii* (F.M. Bailey) C.T. White & Francis	F. No. Qld.	CHCl_3_/EtOH bark	[22]	< 12.5	> 200	> 16	18.7 ± 3.7	> 11
*Erythrina lanceolata* Standl.	Monteverde	CH_2_Cl_2_ bark	[21]	> 200	6.9 ± 1.2	-	-	-
*Erythrina lanceolata* Standl.	Monteverde	EtOH bark	[21]	< 12.5	129.5 ± 3.4	> 10	22.3 ± 3.4	6
*Eugenia monteverdensis* Barrie	Monteverde	acetone bark	b	23.9 ± 2.8	> 200	> 8	20.7 ± 4.5	> 10
*Eugenia* sp. “fine leaf”	Monteverde	acetone bark	[21]	< 12.5	> 200	> 16	< 12.5	> 16
*Euphorbia elata* Brandegee	Monteverde	MeOH bark	[21]	> 200	48.3 ± 3.6	-	-	-
*Euphorbia elata* Brandegee	Monteverde	acetone bark	[21]	> 200	21.6 ± 2.2	-	-	-
*Exothea paniculata* (Juss.) Radlk.	Abaco	acetone bark	b	> 200	49.8 ± 1.8	-	-	-
*Exothea paniculata* (Juss.) Radlk.	Abaco	MeOH bark	b	> 200	> 200	-	-	-
*Exothea paniculata* (Juss.) Radlk.	Monteverde	CHCl_3_ bark	b	< 12.5	> 200	> 16	< 12.5	> 16
*Exothea paniculata* (Juss.) Radlk.	Monteverde	MeOH bark	b	33.2 ± 3.6	159.1 ± 7.5	5	32.3 ± 7.5	5
*Forestiera carthaginense* Donn. Sm.	Monteverde	CHCl_3_/EtOH bark	[21]	> 200	> 200	-	-	-
*Inga sierrae* Britton & Killip	Monteverde	acetone bark	[21]	25.7 ± 1.9	130.6 ± 2.5	5	29.9 ± 5.5	4
*Lonchocarpus oliganthus* F.J. Herm	Monteverde	acetone bark	[21]	20.6 ± 2.6	29.8 ± 4.8	1	-	-
*Lonchocarpus orotinus* Pittier	Monteverde	acetone bark	b	> 200	176.8 ± 5.9	-	-	-
*Macaranga subdentata* Benth.	Paluma	CHCl_3_/EtOH leaf	[20]	> 200	> 200	-	-	-
*Machaerium biovulatum* Micheli	Monteverde	acetone bark	[21]	> 200	120.8 ± 4.5	-	-	-
*Mallotus mollissimus* (Geiseler) Airy Shaw	F. No. Qld.	CHCl_3_/EtOH bark	[22]	20.9 ± 0.3	> 200	> 10	25.2 ± 1.0	> 8
*Mallotus paniculatus* (Lam.) Muell. Arg.	F. No. Qld.	EtOH bark	[22]	< 12.5	> 200	> 16	< 12.5	> 16
*Matelea pseudobarbata* (Pittier) Woodson	Monteverde	EtOH aerial parts	[21]	< 12.5	> 200	> 16	< 12.5	> 16
*Melicope broadbentiana* Bailey	Paluma	CHCl_3_/EtOH bark	[20]	23.7 ± 4.3	79.7 ± 0.3	3	-	-
*Melicope jonesii* T.G. Hartley	F. No. Qld.	CHCl_3_ bark	[22]	< 12.5	35.5 ± 8.6	> 3	33.4 ± 5.0	1
*Melicope rubra* (Lauterb. & K. Schum) T.G. Hartley	F. No. Qld.	EtOH bark	[22]	< 12.5	29.6 ± 0.4	> 2	22.0 ± 2.0	1
*Melicope vitiflora* (F. Muell.) T. Hartley	Paluma	EtOH bark	[20]	> 200	62.6 ± 3.6	-	-	-
*Mucuna urens* (L.) DC.	Monteverde	MeOH bark	[21]	> 200	63.6 ± 4.2	-	-	-
*Myrcianthes* sp. “black fruit”	Monteverde	CHCl_3_ bark	[21]	< 12.5	48.8 ± 2.0	> 4	18.3 ± 4.4	3
*Neea psychotrioides* Donn. Sm.	Monteverde	CHCl_3_ bark	[21]	> 200	> 200	-	-	-
*Neolitsea dealbata* (R. Br.) Merr.	Paluma	EtOH bark	[20]	> 200	> 200	-	-	-
*Ocotea* sp. “los llanos”	Monteverde	acetone bark	b	> 200	> 200	-	-	-
*Octea meziana* C.K. Allen	Monteverde	acetone bark	[21]	> 200	132.1 ± 6.8	-	-	-
*Ormosia cruenta* Rudd	Monteverde	acetone bark	[21]	> 200	198.5 ± 2.1	-	-	-
*Pappea capensis* Eckl. & Zeyh.	Matabeleland	MeOH bark	b	< 12.5	55.6 ± 7.4	> 4	< 12.5	> 4
*Phoradendron* cf. *Flavens* (Sw) Griseb.	Monteverde	CH_2_Cl_2_/EtOH aerial parts	[21]	> 200	> 200	-	-	-
*Phoradendron robustissimum* Eichler	Monteverde	CH_2_Cl_2_/EtOH aerial parts	[21]	44.1 ± 4.9	166.4	4	-	-
*Phoradendron robustissimum* Eichler	Monteverde	CH_2_Cl_2_/EtOH flowers	[21]	> 200	> 200	-	-	-
*Piper aequale* Vahl	Monteverde	acetone leaf	[21]	81.1 ± 1.4	35.9 ± 7.6	0	-	-
*Polyosma alangiacea* F. muell.	F. No. Qld.	CHCl_3_ bark	[22]	60.9 ± 8.9	> 200	> 3	46.2 ± 3.7	> 4
*Psychotria parvifolia* Benth.	Monteverde	acetone bark	b	> 200	> 200	-	-	-
*Quercus insignis* M. Martens & Galeotti	Monteverde	CH_2_Cl_2_ bark	[21]	> 200	161.4 ± 6.3	-	-	-
*Quercus insignis* M. Martens & Galeotti	Monteverde	EtOH bark	[21]	17.8 ± 2.3	> 200	> 11	21.0 ± 3.0	> 10
*Rhynchosia edulis* Griseb.	Monteverde	MeOH bark	[21]	> 200	144.9 ± 1.2	-	-	-
*Rhynchosia resinosa* Hochst. ex Baker	Matabeleland	MeOH root	b	54.1 ± 4.2	> 200	> 4	27.7 ± 5.4	> 7
*Ruyschia phylladenia* Sandwith	Monteverde	CH_2_Cl_2_ aerial parts	[21]	< 12.5	> 200	> 16	22.0 ± 5.9	> 9
*Salacia petenesis* Lundell	Monteverde	EtOH bark	[21]	> 200	35.4 ± 0.8	-	-	-
*Salacia* sp. “liana”	Monteverde	CH_2_Cl_2_ bark	[21]	> 200	79.2 ± 3.8	-	-	-
*Salacia* sp. “liana”	Monteverde	MeOH bark	[21]	>200	91.5 ± 0.3	-	-	-
*Sapium glandulosum* (L.) Morong	Monteverde	acetone bark	[21]	> 200	> 200	-	-	-
*Sapium glandulosum* (L.) Morong	Monteverde	CH_2_Cl_2_ bark	[21]	> 200	73.3 ± 5.6	-	-	-
*Sapium glandulosum* (L.) Morong	Monteverde	EtOH bark	[21]	> 200	> 200	-	-	-
*Sassafras albidum* (Nutt.) Nees	Alabama^h^	CH_2_Cl_2_ bark	b	< 12.5	> 200	> 16	20.5 ± 0.6	> 10
*Sassafras albidum* (Nutt.) Nees	Alabama	EtOAc bark	b	19.4 ± 3.8	> 200	> 10		
*Saurauia montana* Seem	Monteverde	acetone leaf	[21]	> 200	> 200	-	-	-
*Sinclaria polyantha* (Klatt) Rydb.	Monteverde	CHCl_3_/EtOH leaf	[21]	> 200	> 200	-	-	-
*Sorocea trophoides* W.C. Burger	Monteverde	MeOH bark	[21]	> 200	> 200	-	-	-
*Stauranthus perforatus* Leibm.	Monteverde	EtOH bark	[21]	> 200	> 200	-	-	-
*Stemmadenia donnell-smithii* (Rose) Woodson	Monteverde	acetone bark	[21]	< 12.5	> 200	> 16	< 12.5	> 16
*Stenocarpus sinuatus* (Loudon) Endl.	F. No. Qld.	CHCl_3_/EtOH bark	[22]	39.0 ± 1.0	36.8 ± 0.3	1	-	-
*Stockwellia quadrifida* .J. Carr, S.G.M. Carr, & B. Hyland	F. No. Qld.	EtOH bark	[22]	> 200	> 200	-	-	-
*Struthanthus* cf. *oerstedii* (Oliv.) Standl.	Monteverde	CHCl_3_/EtOH leaf	[21]	> 200	> 200	-	-	-
*Styphnolobium monteviridis* M. Sousa & Rudd	Monteverde	MeOH leaf	[21]	> 200	194.8 ± 7.3	-	-	-
*Styphnolobium monteviridis* M. Sousa & Rudd	Monteverde	MeOH bark	[21]	> 200	105.3 ± 7.4	-	-	-
*Symplocos limoncillo* Humb. & Bonpl.	Monteverde	EtOH bark	[21]	> 200	> 200	-	-	-
*Syncarpia glomulifera* (Smith) Niedenzu	Paluma	CHCl_3_ bark	[20]	> 200	> 200	-	-	-
*Syncarpia glomulifera* (Smith) Niedenzu	Paluma	EtOH bark	[20]	> 200	> 200	-	-	-
*Syzygium gustavioides* (F.M. Bailey) B. Hyland	F. No. Qld.	CHCl_3_/EtOH bark	[22]	> 200	> 200	-	-	-
*Tabebuia bahamensis* (Northr.) Britton	Abaco	acetone bark	b	17.4 ± 3.5	> 200	> 11	23.8 ± 5.7	> 8
*Weinmannia pinnata* L.	Monteverde	EtOH bark	[21]	> 200	> 200	-	-	-
*Xanthophyllum octandrum* (F. Muell.) Domin	Paluma	EtOH bark	[20]	33.7 ± 3.6	20.6 ± 0.6	1	-	-
*Zanthoxylum rhoifolium* Lam.	Monteverde	MeOH bark	b	34.2 ± 2.3	122.0 ± 7.0	4	-	-
*Zanthoxylum setulosum* P. Wilson	Monteverde	MeOH bark	[21]	188.9 ± 1.6	> 200	-	-	-
*Zanthoxylum setulosum* P. Wilson	Monteverde	MeOH bark	[21]	> 200	> 200	-	-	-
*Zanthoxylum setulosum* P. Wilson	Monteverde	CHCl_3_ bark	[21]	> 200	> 200	-	-	-
*Zanthoxylum* sp. aff. *Juniperinum* Poepp	Monteverde	EtOH bark	[21]	< 12.5	14.9 ± 2.2	> 1	-	-
*Zanthoxylum veneficum* F.M. Bailey	F. No. Qld.	CHCl_3_/EtOH bark	[22]	> 200	66.9 ± 7.9	-	-	-
Amphotericin B	-	-	-	0.030 ± 0.003	5.8 ± 0.5	193	0.030 ± 0.003	193
Pentamidine	-	-	-	0.37 ± 0.01	11.7 ± 1.7	32	1.3 ± 0.1	9

^a^ Abaco Island, Bahamas. ^b^ Unpublished. ^c^ Monteverde, Costa Rica. ^d^ Paluma, north Queensland, Australia. ^e^ Far north Queensland, Australia. ^f^ Matabeleland, Zimbabwe. ^g^ Navarre, Florida, USA. ^h^ Huntsville, Alabama, USA.

## 4. Conclusions

Of the 115 extracts screened, 25 (21.7%) showed promising activity against *L. amazonensis* promastigotes with low cytotoxic activity. Additional antileishmanial screening against *L. amazonensis* amastigotes revealed ten of these extracts (8.7%) to be considered “hits”, with selectivity indices, *CC*_50_ (macrophage)/*IC*_50_ (amastigotes) greater than 10 that are worthy candidates for further phytochemical exploration: *Conostegia xalapensis* methanol bark extract, *Endiandra palmerstonii* bark extract, *Eugenia monteverdensis* acetone bark extract, *Eugenia* sp. “fine leaf” acetone bark extract, *Exothea paniculata* chloroform bark extract, *Mallotus paniculatus* ethanol bark extract, *Matelea pseudobarbata* ethanol extract, *Quercus insignis* ethanol bark extract, *Sassafras albidum* dichloromethane bark extract, and *Stemmadenia donnell-smithii* acetone bark extract. The good antileishmanial activity coupled with low cytotoxicity to mammalian cells indicates promise in terms of therapeutic index. Phytochemical analyses are currently underway in our laboratories. The results of this study demonstrate the medicinal potential of tropical rainforests and may provide complementary, safe, and affordable therapeutics for treatment of leishmaniasis. A total of 85 extracts were inactive or unspecific, which constitute 74% of tested samples, and reinforces the need to screen large series of natural products to find promising ones.
